# Supplementary material for: A Retrospective Study of Staphylococcus aureus Bacteremia in a Tertiary Hospital and Factors Associated with Mortality
Source: Diagnostics (Basel). 2023 Jun 5;13(11):1975. doi: 10.3390/diagnostics13111975 (PMC10253165; doi:10.3390/diagnostics13111975)
Supplement: Supplementary file 1 [file diagnostics-13-01975-s001.zip › diagnostics-2380792-supplementary.pdf]

diagnostics

MDPI

Table S1. STROBE information for the study regarding methods and the results.

**Methods**	
Study design	Observational, retrospective, descriptive study
Setting	Patients hospitalized in the University Hospital of Heraklion, Greece
Participants	Patients with *Staphylococcus aureus* bacteremia (at least one positive blood culture for this pathogen)
Variables	• Record of outcomes of *S. aureus* bacteremia<br>• Record of demographic characteristics, clinical values, comorbidities, microbiological characteristics, and antimicrobial treatment
Data sources/ measurement	• Data of patients with *S. aureus* derived from the microbiology laboratory<br>• The rest of the patients' data derived from the hard copies and the electronical medical records of the hospital
Study size	Target population: all patients with *S. aureus* bacteremia
Bias	Diligence regarding informing patients' file in hard copies and the electronic medical records<br>Recording demographics and medication regimens<br>Analysis of data regarding the significance
Statistical methods	• Categorical data were analyzed with Fisher's exact test<br>• Continuous variables were compared using the Mann–Whitney U-test for non-normally distributed variables<br>• All tests were two-tailed and a p-value equal or lower than 0.05 was considered significant<br>• Data are presented as numbers (%) for categorical variables and medians (interquartile range) or means (+/- standard deviation) for continuous variables<br>• A linear-regression analysis model was developed to evaluate the effect of several parameters with in-hospital mortality<br>• A multivariate logistic-regression analysis model was developed to evaluate the association of factors identified in the univariate analysis with a p lower than or equal to 0.1 with mortality. For multivariate analysis a p-value equal or lower than 0.05 was considered significant (along with a confidence interval of 95%)
**Results**	
Participants	256 patients were enrolled in the present study
Descriptive data	• Median age of 72 years and 101 (39.5%) were female<br>• Positive blood culture drawn in a medical ward (80.5%), surgical ward (13.5%) and the ICU (6%)<br>• Bacteremia was community-acquired in 49.5%<br>• *S. aureus* was methicillin-resistant in 37.9%<br>• A repeat blood culture was taken in 14.4% after initiation of antimicrobial treatment<br>• Median duration of stay in the hospital was 20 days

Outcome data	• In-hospital mortality was 15.9%
Main results	• Female gender (p=0.0386), higher age (p=0.001), higher McCabe score (p=0.0002), previous antimicrobial use (p=0.0401), presence of a CVC (p=0.0009), neutropenia (p=0.0102), severe sepsis (p<0.0001), septic shock (p<0.0001), and bacteremia by methicillin-resistant *S. aureus* (p=0.03) were positively associated, while monomicrobial bacteremia (p=0.0055) was negatively associated with in-hospital mortality in the univariate regression analysis<br>• The multivariate logistic-regression model identified only severe sepsis (p = 0.05, odds ratio = 12.294 (95% confidence intervals 1.005-150.354) and septic shock (p = 0.007, odds ratio 57.18, 95% confidence intervals 3.051-1,071.664) to be independently positively associated with in-hospital mortality

Table S2. Medical conditions of patients with *Staphylococcus aureus* bacteremia.

**Characteristic**	***N* (%)**
Hypertension	84 (45.7)
Diabetes	59 (32.1)
Kidney disease	46 (25)
Smoking	33 (17.9)
Malignancy	31 (16.8)
Chronic pulmonary disease	30 (16.3)
Coronary artery disease	26 (14.1)
Hypothyroidism	19 (10.3)
Rheumatologic disease	18 (9.8)
Congestive heart failure	17 (9.2)
Dementia	17 (9.2)
Peripheral vascular disease	16 (8.7)
Cerebrovascular disease	14 (7.6)
